# Are we all in this together? Longitudinal assessment of cumulative adversities by socioeconomic position in the first 3 weeks of lockdown in the UK

**DOI:** 10.1136/jech-2020-214475

**Published:** 2020-09-01

**Authors:** Liam Wright, Andrew Steptoe, Daisy Fancourt

**Affiliations:** 1 Department of Epidemiology and Public Health, University College London, London, UK; 2 Department of Behavioural Science and Health, University College London, London, UK

**Keywords:** EPIDEMIOLOGY, PUBLIC HEALTH, MENTAL HEALTH, Cohort studies, PSYCHOSOCIAL FACTORS

## Abstract

**Background:**

Despite media claims that coronavirus disease 2019 (COVID-19) is uniting societies and countries in shared experience, there has been concern that the pandemic is in fact exposing and widening existing inequalities within societies. Data have shown these differences for cases and fatalities, but data on other types of adversities are lacking. Therefore, this study explored the changing patterns of adversity relating to the COVID-19 pandemic by socioeconomic position (SEP) during the early weeks of lockdown in the UK.

**Methods:**

Data were from 12 527 UK adults in the University College London COVID-19 Social Study (a panel study that involves online weekly data collection from participants during the COVID-19 pandemic). We analysed data collected from 25 March to 14 April 2020. The sample was well-stratified and weighted to population proportions of gender, age, ethnicity, education and country of living. We used Poisson and logit models to assess 10 different types of adverse experiences depending on an index of SEP over time.

**Results:**

There was a clear gradient across the number of adverse events experienced each week by SEP. This was most clearly seen for adversities relating to finances (including loss of employment and cut in income) and basic needs (including access to food and medications) but less for experiences directly relating to the virus. Inequalities were maintained with no reductions in discrepancies between socioeconomic groups over time.

**Conclusions:**

There were clear inequalities in adverse experiences during the COVID-19 pandemic in the early weeks of lockdown in the UK. Results suggest that measures taken to try to reduce such adverse events did not go far enough in tackling inequality.

## INTRODUCTION

Over the past few weeks, there have been claims in the media that coronavirus disease 2019 (COVID-19) is uniting societies and countries in shared experience: ‘we are all in this together’. However, scientific papers are beginning to emerge arguing that COVID-19 is disproportionately affecting vulnerable populations. Much of this research has focused on inequalities in cases and fatalities, citing challenges for more disadvantaged groups due to individuals facing difficulties in accessing healthcare in certain countries, being less able to adhere to protective social distancing measures due to living in more overcrowded areas, having a higher burden of pre-existing diseases and risk factors, being disproportionally affected by misinformation and miscommunication, and not being able to afford to lose income from missing work.^1–^ ^4^ Nevertheless, there has also been concern that the virus could expose and widen existing inequalities within societies.^2^ ^5–^ ^7^ This is particularly problematic as it could trigger a vicious cycle of increasing inequalities that weaken economic structures within societies and also exacerbate the spread of the virus, leading to the labelling of COVID-19 as a ‘pandemic of inequality’.^4 5 7^

Studies from previous epidemics such as severe acute respiratory syndrom (SARS), Middle East respiratory syndrome (MERS) and Ebola have suggested that people can experience a range of adversities during and in the aftermath of epidemics.^8^ These can include adversities related to the virus itself (such as infection or bereavement), as well as challenges meeting basic needs (such as access to food, medication and accommodation),^9–^ ^11^ and the experience of financial loss (including loss of employment and income).^11–^ ^16^ The wider health literature suggests that people from lower socioeconomic backgrounds are less resilient to shocks such as ill-health, experiencing greater financial burden, and hardship.^17^ This suggests there is likely to be a social gradient in these experiences during COVID-19, but so far there has been limited empirical investigation of inequalities in experience of adversity during the pandemic. Nevertheless, these experiences of burden and hardship are vital to understand as studies of previous epidemics have found a relationship between experience of adversity and psychological consequences including post-traumatic stress and depression.^16^ This echoes wider literature on the strong relationship between adversities relating to finances, basic needs, and ill-health, and poor mental and physical health outcomes.^18–^ ^21^

Therefore, this study explored the changing patterns of adversity relating to the COVID-19 pandemic by socioeconomic position (SEP) during the first few weeks of lockdown in the UK. We focused on three types of adversity: (1) financial stressors (loss of work, partner’s loss of work, cut in household income or inability to pay bills), (2) challenges relating to basic needs (including food, medications and accommodation) and (3) experience of the virus itself (including contracting the virus, a close person being hospitalised and a close person dying). We sought to explore the nature of the relationship between SEP and (1) number of adversities experienced, (2) type of adversity experienced, and (3) how the relationship evolved over the first 3 weeks of lockdown.

## METHODS

### Participants

Data were drawn from the University College London (UCL) COVID-19 Social Study—a large panel study of the psychological and social experiences of over 70 000 adults (aged 18+) in the UK during the COVID-19 pandemic. The study commenced on 21 March 2020, with recruitment ongoing. The study involves online weekly data collection from participants during the COVID-19 pandemic in the UK. While not random, the study has a well-stratified sample that was recruited using three primary approaches. First, snowballing was used, including promoting the study through existing networks and mailing lists (including large databases of adults who had previously consented to be involved in health research across the UK), print and digital media coverage, and social media. Second, more targeted recruitment was undertaken focusing on (1) individuals from a low-income background, (2) individuals with no or few educational qualifications, and (3) individuals who were unemployed. Third, the study was promoted via partnerships with third sector organisations to vulnerable groups, including adults with pre-existing mental illness, older adults and carers. The study was approved by the UCL Research Ethics Committee (12467/005) and all participants gave informed consent.

Questionnaire items related to newly experienced adversities were available from 25 March 2020— 1 day after legal enforcement of lockdown commenced. We used data from the 3 weeks following this date (25 March–14 April 2020), limiting our analysis to a balanced panel of participants who were interviewed in all of these weeks (n=14 309; 58.7% of individuals interviewed between 25 and 31 March 2020). We excluded participants with missing data on any variable used in this study (n=1782; 12.45% of balanced panel; 3.21% missing weights, 9.67% missing SEP measures and 0.01% missing outcome measure). This provided a final analytical sample of 12 527 participants.

### Measures

#### Adversities

Questions on 10 separate adversities were recorded each week. Four of these assessed financial adversity: whether participants had lost their job or been unable to work, their partner had lost their job or was unable to work, they had experienced a major cut in household income (data available from the second week) or they had been unable to pay bills. Three questions assessed adversity relating to basic needs: whether participants had lost their accommodation, they had been unable to access sufficient food, or they had been unable to access required medication. Finally, three questions assessed adversity directly relating to the virus: whether in the past week the participant had suspected or diagnosed COVID-19, somebody close to them was hospitalised, or they had lost somebody close to them. We constructed a weekly total adversity measure by summing the number of adversities present in a given week (range 0–10). For adversities that were considered to be cumulative (ie, once experienced in 1 week, their effects would likely last into future weeks), we also counted them on subsequent waves after they had first occurred. This applied to experiencing suspected/diagnosed COVID-19, the loss of work for a participant or their partner, a major cut in household income, and the loss of somebody close to the participant.

#### Socioeconomic position

We measured SEP using five variables collected at baseline interview: (1) annual household income (<£16 000, £16 000–£30 000, £30 000–£60 000, £60 000–£90 000, £90 000+), (2) highest qualification (General Certificate of Secondary Education (GCSE) or lower (qualifications at age 16), A-Levels or vocational training (qualifications at age 18), undergraduate degree, postgraduate degree), (3) employment status (employed, inactive and unemployed), (4) housing tenure (own outright, own with mortgage, rent/live rent-free) and (5) household overcrowding (binary: >1 person per room). From these variables, we constructed a *Low SEP* index measure by counting indications of low SEP (income <£16 000, educational qualifications of GCSE or lower, unemployed, living in rented or rent-free accommodation, and living in overcrowded accommodation), collapsing into 0, 1 and 2+ indications of low SEP to attain adequate sample sizes for each category.

#### Covariates

To account for broad demographic differences that could confound the association between SEP and adversity experiences, we also included variables for gender (male, female), age (18–24, 25–34, 35–49, 50–64, 65+), marital status (cohabiting with partner, living away from partner, single, divorced/widowed) and ethnicity (white, non-white).

### Analysis

We assessed experienced adversities according to SEP by estimating Poisson models for each of the 3 weeks separately. First, we extracted the predicted number of adversities according to SEP using average marginal effects and plotted the estimates to test whether social gradients were present and whether they changed in size by week. Second, we repeated this exercise for each adversity separately by estimating logit models for each adversity and each week of data. Analyses were adjusted for age, gender, ethnicity and marital status. Third, we compared estimated differences in the prevalence of adversities between highest and lowest SEP groups in weeks 1 and 3 to explore if there was any evidence of change in inequalities over time. To account for the non-random nature of the sample, all data were weighted to the proportions of gender, age, ethnicity, education and country of living obtained from the Office for National Statistics.^22^

We carried out several sensitivity analyses to test the robustness of our results. First, to test whether findings were an artefact of our chosen statistical method, we repeated the Poisson regressions using negative binomial and zero-inflated Poisson models. Second, to test whether findings were driven by our type of SEP index, we repeated analyses using the individual SEP variables directly and deriving an alternative SEP measure using confirmatory factor analysis (CFA). The CFA used weighted least square mean, and given the discrete nature of the SEP indicators, the variance adjusted (WLSMV) estimator was implemented. The root mean square error of approximation of the CFA model was 0.08, indicating an adequate fit.^23^ We split the latent factor into five groups using natural breaks in the factor values. Third, as the reporting of COVID-19 symptoms is likely biased due to asymptomatic cases or differences in recognition of symptoms, the latter of which is likely to be related to health literacy and thus to SEP, we excluded suspected/diagnosed COVID-19 from the total adversity measure. Finally, as several of the adversities considered here are related to loss of employment or paid work, we repeated each analysis restricting the sample to adults who were employed at baseline.

## RESULTS

### Descriptive statistics

Descriptive statistics for the sample are shown in table 1. Once weighting had been applied, our sample closely matched population averages on gender, age, ethnicity, education and country of living. Unweighted figures are shown in Supplementary table 1.

**Table 1:** Descriptive sample statistics weighted according to ONS data

	Variable	n (%)
Low SEP index: number of indicators of low SEP	0	5517.5 (44)
	1	4227.3 (33.7)
	2+	2782.2 (22.2)
Household income	£90 000+	1129.2 (9)
	£60 000–£90 000	1689.5 (13.5)
	£30 000–£60 000	4009.5 (32)
	£16 000–£30 000	3315.5 (26.5)
	<£16 000	2383.4 (19)
Employment status	Employed	7371.4 (58.8)
	Inactive	4974.6 (39.7)
	Unemployed	181 (1.4)
Highest qualification	Postgraduate	2297.5 (18.3)
	Undergraduate	2863.9 (22.9)
	A-Level or vocational	3853.7 (30.8
	GCSE or lower	3511.9 (28)
Household tenure	Own outright	4491.2 (35.9)
	Own mortgage	3971.4 (31.7)
	Rent	4064.3 (32.4)
Overcrowded accommodation	Not overcrowded	11 899.7 (95)
	Overcrowded	627.3 (5)
Gender	Male	6047.4 (48.3)
	Female	6479.6 (51.7)
Age	18–24	601.4 (4.8)
	25–34	1690.9 (13.5)
	35–49	3122.8 (24.9)
	50–64	4024.4 (32.1)
	65+	3087.6 (24.6)
Ethnicity	White	11 227 (89.6)
	Non-white	1300 (10.4)
Marital status	Living with partner	7690.6 (61.4)
	Living without partner	840.2 (6.7)
	Single	2282.7 (18.2)
	Divorced or widowed	1713.5 (13.7)

All descriptive statistics are weighted by age group, gender and education level, which accounts for fractions.

GCSE, General Certificate of Secondary Education; ONS, Office for National Statistics; SEP, socioeconomic position.

10.1136/jech-2020-214475.supp1 Supplementary data

The prevalence of adversities overall and by week is shown in table 2. Average number of adversities increased over the follow-up period, as did variability. Within the first 3 weeks, one in six participants reported a major cut in ousehold income and either them or their partner losing work. Numbers experiencing symptoms of COVID-19, or losing people close to them also increased. Conversely, numbers of participants being unable to access food or medication fell week by week.

**Table 2:** Weighted descriptive statistics, total and individual adversities

Variable Mean (SD)/n (%)	Overall	Week 1	Week 2	Week 3
Number of adversities	0.56 (0.9)	0.41 (0.73)	0.6 (0.92)	0.66 (1)
Lost work	1235.5 (9.9%)	1030.4 (8.2%)	1291.6 (10.3%)	1384.5 (11.1%)
Partner lost work	797.2 (6.4%)	643.5 (5.1%)	823.1 (6.6%)	925.1 (7.4%)
Major cut in household income	1869.6 (14.9%)	.*	1649.7 (13.2%)	2089.4 (16.7%)
Unable to pay bills	402.8 (3.2%)	362.7 (2.9%)	429.4 (3.4%)	416.4 (3.3%)
Lost accommodation	7.6 (0.1%)	16.1 (0.1%)	4.4 (<0.1%)	2.4 (<0.1%)
Unable to access sufficient food	861.7 (6.9%)	1263.1 (10.1%)	766.4 (6.1%)	555.6 (4.4%)
Unable to access required medication	413.6 (3.3%)	460.3 (3.7%)	395.9 (3.2%)	384.7 (3.1%)
Somebody close is ill in hospital	305.1 (2.4%)	148.3 (1.2%)	343.4 (2.7%)	423.6 (3.4%)
Lost somebody close to them	259.9 (2.1%)	87.5 (0.7%)	249.7 (2%)	442.4 (3.5%)
Suspected or diagnosed with COVID-19	1471 (11.7%)	1171.7 (9.4%)	1542 (12.3%)	1699.3 (13.6%)

*Data for cut in household income were not available in week 1.

All descriptive statistics are weighted, which accounts for fractions.

COVID-19, coronavirus disease 2019.

### Adversity by SEP

**Figure 1:**
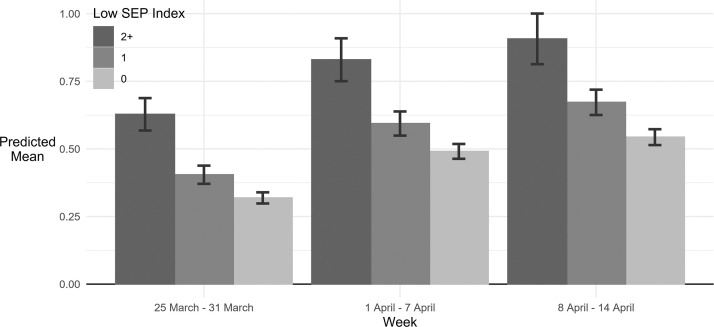
Predicted mean number of adversities experienced by week and SEP, derived from fully adjusted Poisson model. NB dates show the week in which adversities were reported, with reporting being on experiences in the past 7 days. SEP, socioeconomic position.

When applying our low SEP index, the number of adverse events experienced each week showed a clear social gradient (figure 1). Regression results showed a significant difference in the number of adverse events according to the SEP index score among those with scores of 1 and 2+ compared with those with scores of 0 (Supplementary Table 2). When comparing the change in experience in adversities over time by SEP, these inequalities were maintained each week, with no decreases evident over time (Supplementary Table 4).

**Figure 2:**
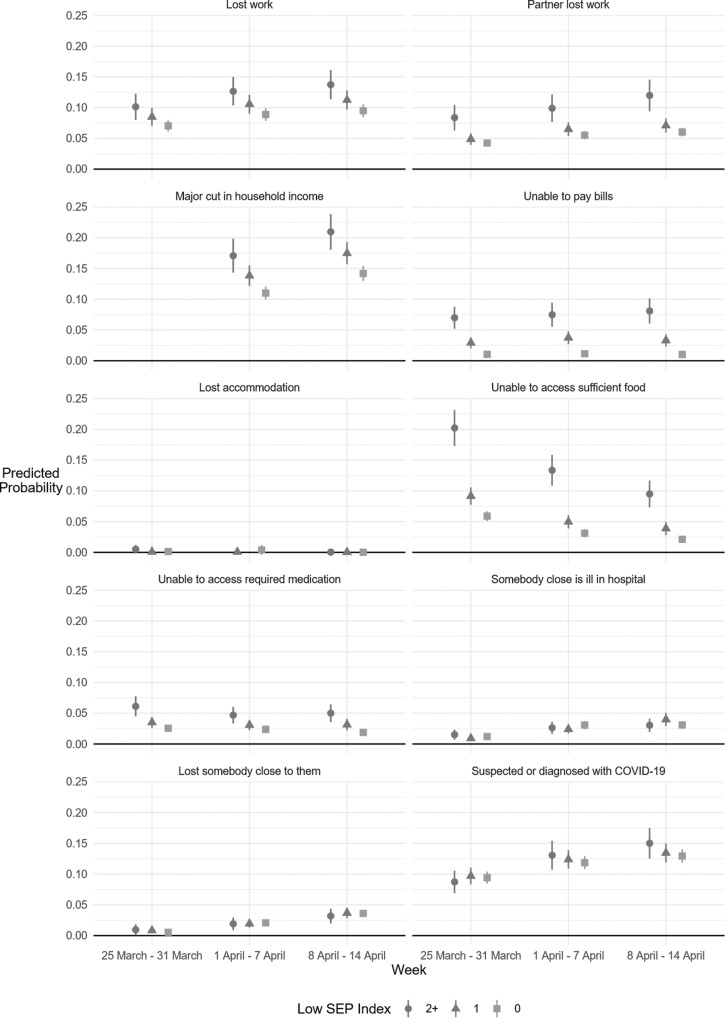
Predicted probability of experiencing specific adversities by week and SEP, from fully adjusted logit models. NB dates show the week in which adversities were reported, with reporting being on experiences in the past 7 days. SEP, socioeconomic position.

When exploring the patterns for each type of adversity individually, there was a clear social gradient across all financial measures and across factors relating to basic needs (figure 2). People of lower SEP were 1.5 times more likely to experience loss of work compared with people of higher SEP, and their partners were twice as likely to experience loss of work (Supplementary Table 3). They were also 7.2 times more likely to be unable to pay bills in week 1 (rising to 8.7 times more likely by week 3), 4.1 times more likely to be unable to access sufficient food in week 1 (rising to 4.9 times more likely be week 3) and 2.5 times more likely to be unable to access required medication. However, there was little evidence of a gradient in experiences directly relating to the virus, with no significant differences between groups. In comparing the change in experience of each specific adversity over time by SEP, the inequalities present in each individual adversity were maintained each week, with no evidence of improvement over time (Supplementary Table 4).

### Sensitivity analyses

When using alternative regression analyses, results were materially unaffected (Supplementary Figure 1), as were results when using CFA rather than our low SEP index (Supplementary Figures 2 and 3). When excluding suspected/diagnosed COVID-19 from the total adversity measure, results showed no meaningful differences (Supplementary Figure 4). Similarly, when restricting the analysis to those employed at baseline, results were qualitatively similar but with a stronger social gradient (Supplementary Figure 5).

## DISCUSSION

This study explored the patterns of adversities in the early weeks of lockdown in the UK due to COVID-19, showing a clear social gradient in experiences. This gradient was evident across the overall number of adversities experienced and specifically across financial stressors and challenges relating to basic needs (including food, medications and accommodation). Inequalities were maintained with no reductions in differences between socioeconomic groups over time.

Notably, this experience of inequalities in financial stressors occurred in the wake of measures announced by government and banks in the UK such as mortgage holidays and furlough schemes aimed at reducing the financial shocks of COVID-19.^24^ While these financial measures implemented may have reduced the discrepancy in experiences between the wealthiest and poorest to a certain extent (it is not possible to test what the alternative scenario might have been), the data presented here show that they did not remove it. This may be because benefits of the schemes did not come into effect immediately within the first month of lockdown (eg, for receipt of furlough payments to be made) or it may indicate that measures were insufficient and individuals of lower SEP still experienced greater financial burden during the pandemic. Even if these initial financial shocks are reduced over time as schemes come into effect and as more measures are taken, they are still concerning, given the well-researched link between experience of adversities and poor mental health outcomes, poor physical health outcomes and suicides.^18–^ ^21^ In planning ahead for anticipated upcoming stages in the fallout from the pandemic, such as a possible future recession, this suggests that more steps need to be taken urgently to reduce further adverse effects for individuals of lower SEP before further negative effects occur.^18^ Further, in terms of preparedness for future pandemics, these results suggest that even more ambitious measures are required early to reduce immediate financial shocks if efforts are to be made to try to avoid widening economic disparities.

Our findings were related to access to basic needs such as food substantiate concerns voiced by academic-practitioners working in food insecurity, food systems and inequality early in the outbreak of COVID-19.^25^ While the data presented here may suggest that although challenges in accessing food decreased in the early weeks following lockdown being implemented in the UK, inequalities in that access remained. It is clearly important that such inequalities are addressed, as there is the potential for both second waves of the virus that might trigger repeat lockdowns, and for further challenges in the functioning of food systems. Planning for the potential of future pandemics should consider how such inequalities could be reduced through early implementation of interventions such as further financial and business support to low-income households, to food charities and food banks, to food producers and to supermarkets, shops and delivery companies.^25^

It is notable that the findings presented here did not show such a clear gradient in experiences of the virus itself within the UK. There is evidence of patterns of inequality in the experience of symptoms of COVID-19 in other literature.^1–^ ^4^ However, given that many cases of the virus are asymptomatic, and low levels of population testing mean that exact infections rates cannot be estimated, our data cannot be taken to represent actual inequalities in cases. Differences in recognition of symptoms are likely to be related to health literacy and thus to SEP, and so may also have affected analyses. Moreover, our questions about experience of bereavement due to COVID-19 or a close family member being hospitalised were asked early in the pandemic when prevalence was low. Our study may have been underpowered to detect clear effects. This also applies to losing accommodation, which occurred for less than 0.2% of the sample. Therefore, our findings do not necessarily imply an absence of inequalities for these experiences and it remains to be seen if inequalities do start to emerge over time. It is also likely that this finding will vary by country depending on the measures taken to reduce the spread of the virus.

This study has several strengths, including its large sample size, its longitudinal tracking of participants and its rich inclusion of measures on socioeconomic factors and experienced adversities during COVID-19. However, there are several limitations. The study is not nationally representative, although it does have good stratification across all major socio-demographic groups and analyses were weighted on the basis of population estimates of core demographics (gender, age, ethnicity, education and country of living). While the recruitment strategy included deliberately targeting individuals of low educational attainment and low household income groups, it is possible that more extreme experiences were not adequately captured. So the inequalities shown in this paper may be underestimations. Further, individuals experiencing particularly high levels of adversity may have withdrawn from the study early, and therefore not been included in our longitudinal sample in these analyses. We lacked follow-up data for 40% of participants (although this does not reflect a drop-out rate for the study as some participants have continued to provide data since, merely outside the window of the dates we focused on for these analyses). Although our use of survey weights may have partly guarded against the effects of selective dropout, it is nonetheless possible that our data present underestimations of inequalities. Additionally, this paper focused exclusively on adversities relating to finances, basic needs and experience of the virus. However, other inequalities have also been noted such as in educational opportunities for children during school closures.^26^ These remain to be explored further in future studies. Finally, our study used two different SEP indices and further tested specific aspects of SEP in sensitivity analyses, but we restricted measurement of SEP to a finite list of factors. Other measures of SEP such as social status or area deprivation and how they relate to adversities experienced remain to be explored further.

The results presented here suggest that there were clear inequalities in adverse experiences during the COVID-19 pandemic in the early weeks of lockdown in the UK. This is notable given that several measures were taken to try to reduce such adverse events, and suggests that such measures did not go far enough in tackling inequality. Further, it is likely that such inequalities in experience will be even greater in low-income countries as the pandemic continues.^7^ The findings from this paper therefore support calls for each country to continually assess which members of society are vulnerable throughout the COVID-19 pandemic to take action to support those at highest risk, and also for planning for future pandemics to include more extensive measures to reduce disproportionate experiences of adversity among lower socioeconomic groups.^7^

**What is already known on this subject**

- A recently published rapid review of the literature on the effects of isolation and quarantine suggested that people can experience a range of adversities during and in the aftermath of the epidemic. These can include adversities related to the virus itself (such as infection or bereavement), as well as challenges meeting basic needs (such as access to food, medication and accommodation), and the experience of financial loss. There has been concern that the COVID-19 pandemic could expose and widen existing inequalities within societies; yet, there have been no empirical analyses.

**What this study adds**

- This study confirms that there was a clear gradient across the number of adverse events experienced each week by SEP during lockdown in the UK. This was most clearly seen for adversities relating to finances and basic needs (including access to food and medications) but less for experiences directly relating to the virus. The findings from this paper suggest that individuals of lower SEP are experiencing more adverse events due to COVID-19 and supports calls for each country to continually assess which members of society are vulnerable throughout the COVID-19 pandemic to take action to support those at highest risk.
