# Supplementary material for: Are we all in this together? Longitudinal assessment of cumulative adversities by socioeconomic position in the first 3 weeks of lockdown in the UK
Source: J Epidemiol Community Health. 2020 Sep 1;74(9):683–8. doi: 10.1136/jech-2020-214475 (PMC7298206; doi:10.1136/jech-2020-214475)
Supplement: Supplementary data [file jech-2020-214475s001.pdf]

Supplementary Material

Supplementary Table 1: Descriptive sample statistics (unweighted)

	Variable	n (%)
Low SEP Index: number of indicators of low SEP	0	7608 (60.7%)
	1	3386 (27%)
	2+	1533 (12.2%)
Household income	£90k+	1618 (12.9%)
	£60k - £90k	2126 (17%)
	£30k - £60k	4376 (34.9%)
	£16k - £30k	2806 (22.4%)
	<£16k	1601 (12.8%)
Employment status	Employed	8441 (67.4%)
	Inactive	3949 (31.5%)
	Unemployed	137 (1.1%)
Highest qualification	Postgraduate	4122 (32.9%)
	Undergraduate	5094 (40.7%)
	A-Level or Vocational	1907 (15.2%)
	GCSE or Lower	1404 (11.2%)
Household tenure	Own Outright	4505 (36%)
	Own Mortgage	4773 (38.1%)
	Rent	3249 (25.9%)
Overcrowded accommodation	Not Overcrowded	12059 (96.3%)
	Overcrowded	468 (3.7%)
Gender	Male	3105 (24.8%)
	Female	9422 (75.2%)
Age	18-24	252 (2%)
	25-34	1733 (13.8%)
	35-49	3874 (30.9%)
	50-64	4178 (33.4%)
	65+	2490 (19.9%)
Ethnicity	White	11953 (95.4%)
	Non-White	574 (4.6%)
Marital status	Living with partner	8096 (64.6%)
	Living without partner	763 (6.1%)
	Single	2019 (16.1%)
	Divorced or Widowed	1649 (13.2%)

Supplementary Table 2: Poisson models assessing total number of stressors by SEP

	**Week 1**<br>**IRR [95%CI]**	**Week 2**<br>**IRR [95%CI]**	**Week 3**<br>**IRR [95%CI]**
**Low SEP index: Unadjusted**			
0	REF	REF	REF
1	1.23<br>[1.11; 1.37]	1.18<br>[1.07; 1.29]	1.20<br>[1.09; 1.31]
2+	1.86<br>[1.66; 2.09]	1.61<br>[1.44; 1.79]	1.57<br>[1.40; 1.76]
**Low SEP index: Adjusted for age, gender, ethnicity and marital status**			
0	REF	REF	REF
1	1.27<br>[1.14; 1.41]	1.21<br>[1.10; 1.33]	1.24<br>[1.13; 1.35]
2+	1.97<br>[1.75; 2.21]	1.69<br>[1.51; 1.89]	1.67<br>[1.48; 1.88]

Supplementary Table 3: Logit models assessing total number of stressors by SEP (reference category Low SEP Index = 0)

Variable	Low SEP Index	Week 1	Week 2	Week 3
Lost work	1	1.23 [0.97, 1.55]	1.22 [0.99, 1.5]	1.21 [0.99, 1.48]
	2+	**1.5 [1.13, 1.98]**	**1.5 [1.17, 1.93]**	**1.53 [1.2, 1.95]**
Partner lost work	1	1.16 [0.9, 1.49]	1.2 [0.95, 1.51]	1.2 [0.96, 1.5]
	2+	**2.12 [1.55, 2.91]**	**1.94 [1.44, 2.62]**	**2.22 [1.66, 2.96]**
Major cut in household income	1		**1.31 [1.09, 1.57]**	**1.3 [1.1, 1.53]**
	2+		**1.7 [1.34, 2.16]**	**1.64 [1.32, 2.04]**
Unable to pay bills	1	**2.87 [1.78, 4.63]**	**3.38 [2.21, 5.15]**	**3.33 [2.14, 5.16]**
	2+	**7.21 [4.55, 11.42]**	**7.07 [4.62, 10.82]**	**8.7 [5.66, 13.39]**
Lost accommodation	1	0.69 [0.1, 4.61]	0.14 [0.01, 1.85]	3.45 [0.34, 35.4]
	2+	3.61 [0.46, 28.34]		3.58 [0.24, 52.75]
Unable to access sufficient food	1	**1.62 [1.29, 2.02]**	**1.62 [1.18, 2.23]**	**1.86 [1.26, 2.75]**
	2+	**4.12 [3.24, 5.24]**	**4.87 [3.53, 6.72]**	**4.89 [3.32, 7.19]**
Unable to access required medication	1	1.39 [0.99, 1.95]	1.31 [0.89, 1.92]	**1.71 [1.13, 2.58]**
	2+	**2.5 [1.76, 3.55]**	**2.03 [1.35, 3.06]**	**2.78 [1.83, 4.23]**
Somebody close is ill in hospital	1	0.76 [0.46, 1.26]	0.78 [0.55, 1.11]	1.3 [0.92, 1.84]
	2+	1.26 [0.71, 2.22]	0.85 [0.55, 1.32]	0.98 [0.64, 1.5]
Lost somebody close to them	1	1.53 [0.78, 3.02]	0.93 [0.64, 1.37]	1.02 [0.76, 1.37]
	2+	1.76 [0.61, 5.04]	0.92 [0.5, 1.67]	0.88 [0.57, 1.35]
Suspected or diagnosed with COVID-19	1	1.03 [0.85, 1.25]	1.05 [0.88, 1.25]	1.05 [0.88, 1.24]
	2+	0.92 [0.71, 1.19]	1.12 [0.88, 1.42]	1.19 [0.95, 1.49]

Supplementary Table 4: Random effects Poisson and logit models with interaction terms between SEP and week exploring changes in inequalities between weeks 1 and 3

**Variable**	**Difference in inequalities from weeks 1-3**
Number of stressors	0.086 [-0.07, 0.241]
Lost work	0.014 [-0.039, 0.067]
Partner lost work	0.052 [-0.018, 0.122]
Unable to pay bills	-0.008 [-0.075, 0.06]
Lost accommodation	-0.006 [-0.027, 0.015]
Unable to access sufficient food	-0.058 [-0.125, 0.009]
Unable to access required medication	0.004 [-0.029, 0.036]
Somebody close is ill in hospital	-0.004 [-0.019, 0.012]
Lost somebody close to them	-0.006 [-0.018, 0.006]
Suspected or diagnosed with COVID-19	0.023 [-0.006, 0.051]

Supplementary Figure 1: Predicted mean number of adversities experienced by week and SEP, derived from fully adjusted (a) negative binomial and (b) zero-inflated Poisson models.

A: Negative Binomial Model

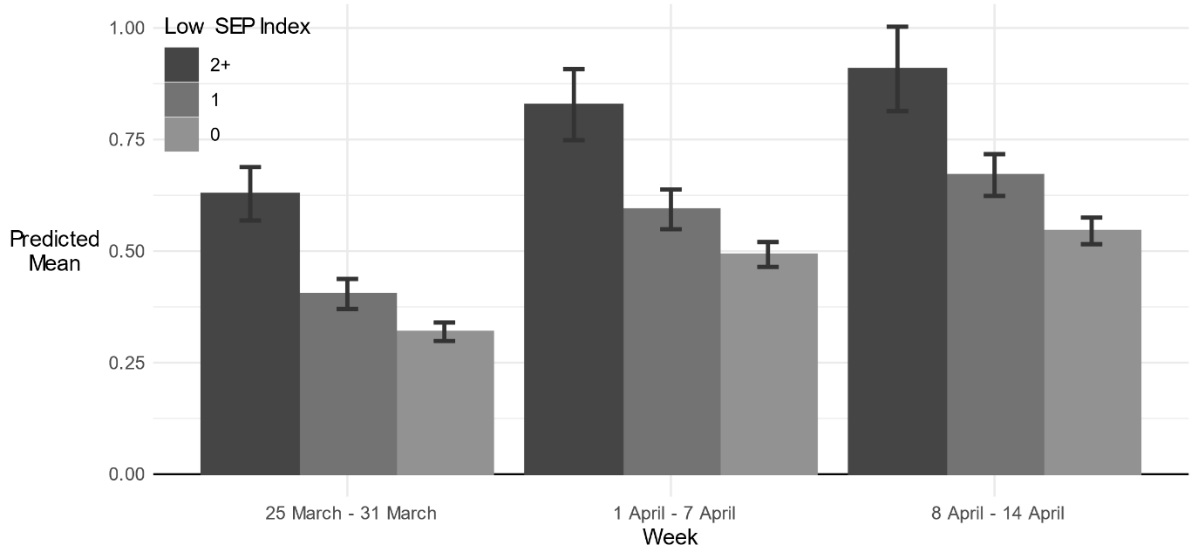

B Zero-Inflated Poisson Model

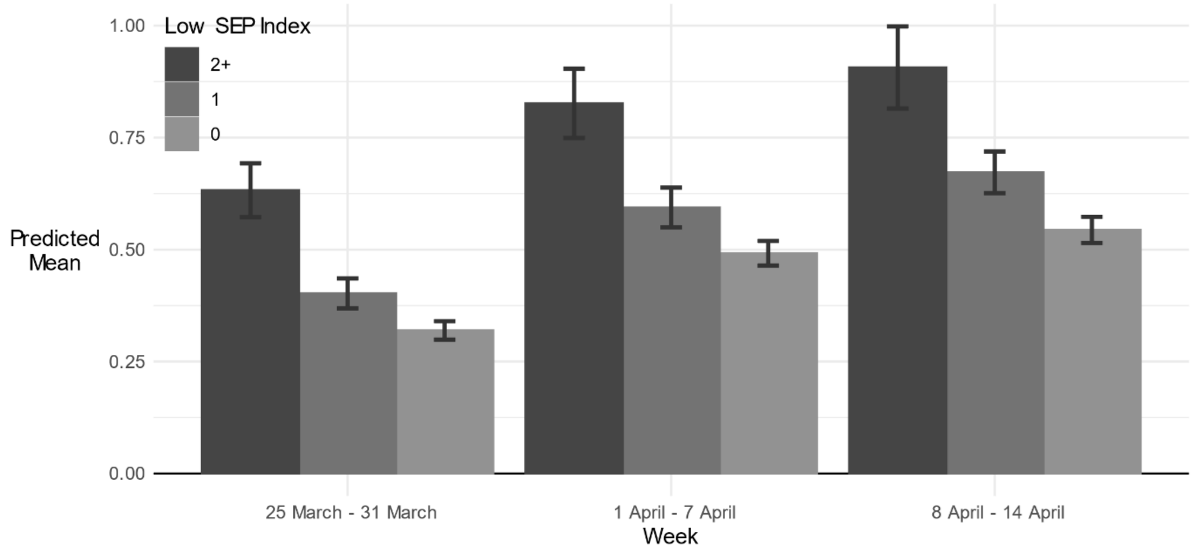

Note: Dates show the week in which adversities were reported, with reporting being on experiences in the past 7 days.

Supplementary Figure 2: Predicted mean number of adversities experienced by week and SEP using Confirmatory Factor Analysis, derived from fully adjusted Poisson model.

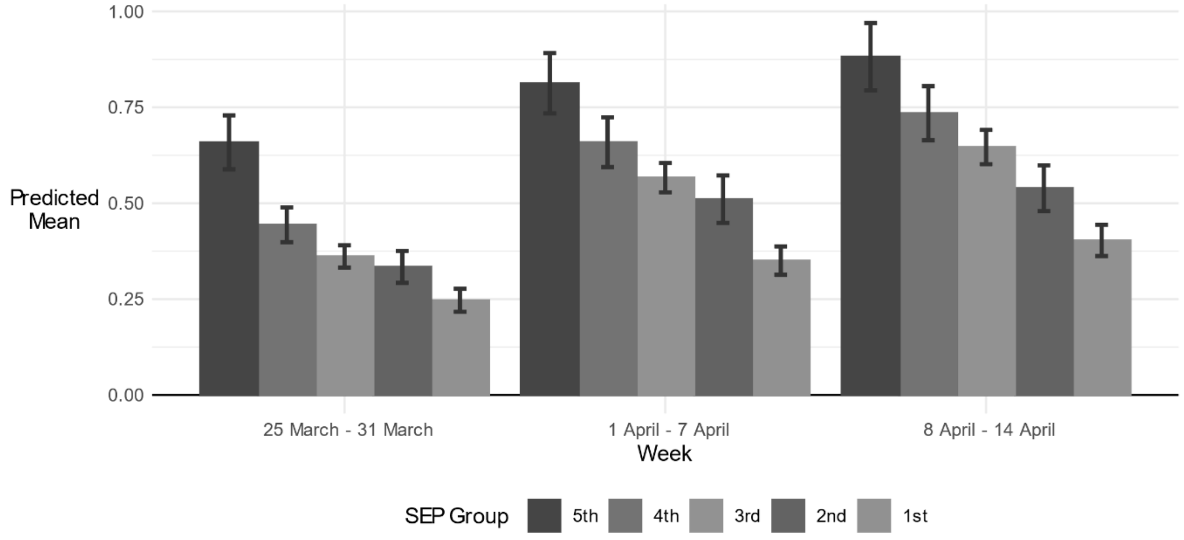

Note: 1st = highest SEP quintile, while 5th = lowest SEP quintile. Groups were determined by natural breaks in the factor values. Group 1: 8.9% of the sample, group 2 13.5%, group 3 31.9%, group 4 26.6%, group 5 %19.0%. Dates show the week in which adversities were reported, with reporting being on experiences in the past 7 days.

Supplementary Figure 3: Predicted probability of experiencing specific adversities by week and SEP using Confirmatory Factor Analysis, from fully adjusted logit models.

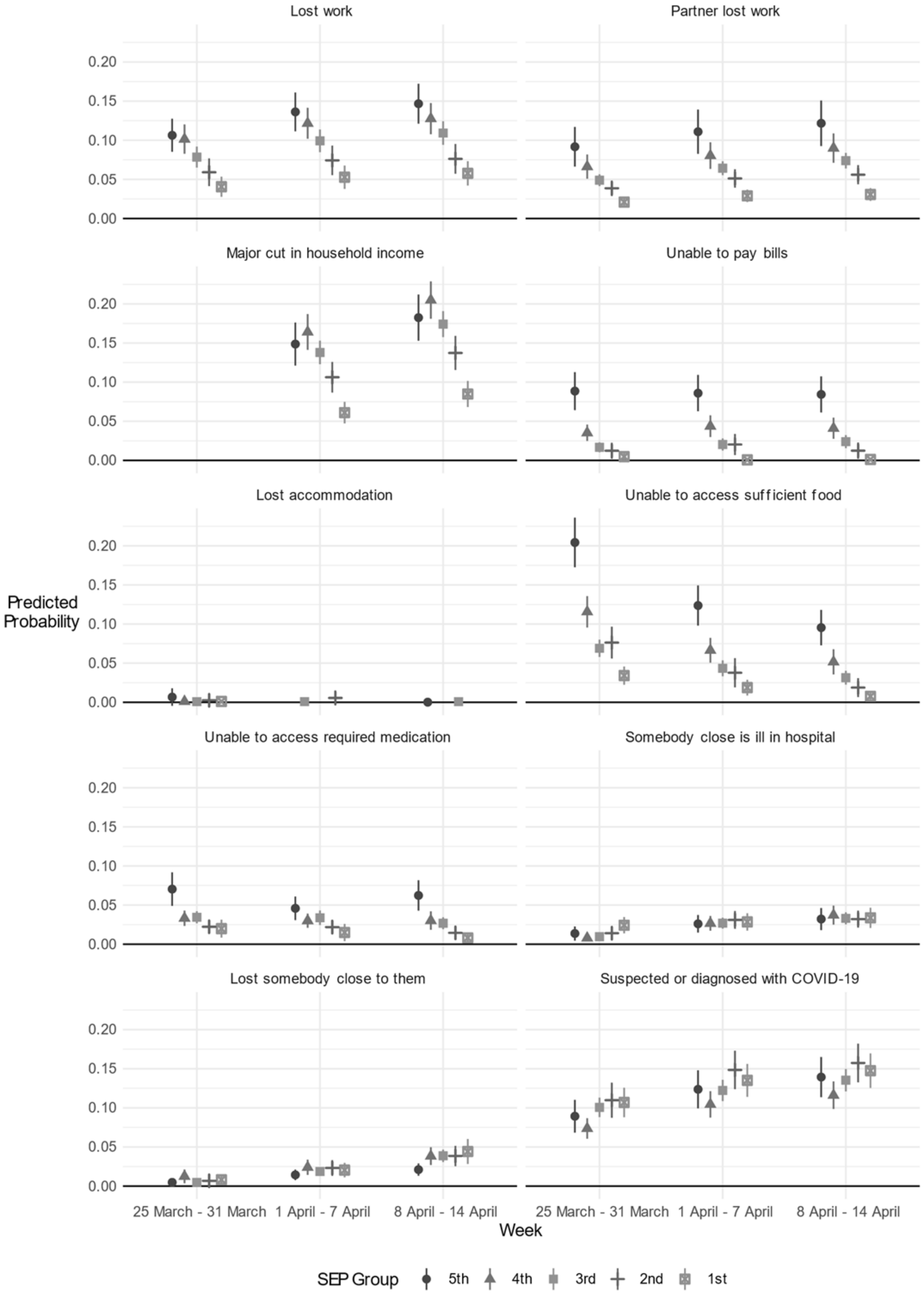

Note: 1st = highest SEP quintile, while 5th = lowest SEP quintile. Groups were determined by natural breaks in the factor values. Group 1: 8.9% of the sample, group 2 13.5%, group 3 31.9%, group 4 26.6%, group 5 %19.0%. Dates show the week in which adversities were reported, with reporting being on experiences in the past 7 days.

Supplementary Figure 4: Predicted probability of experiencing specific adversities by week and SEP, from fully adjusted logit models excluding suspected/diagnosed COVID-19 from the list of adversities

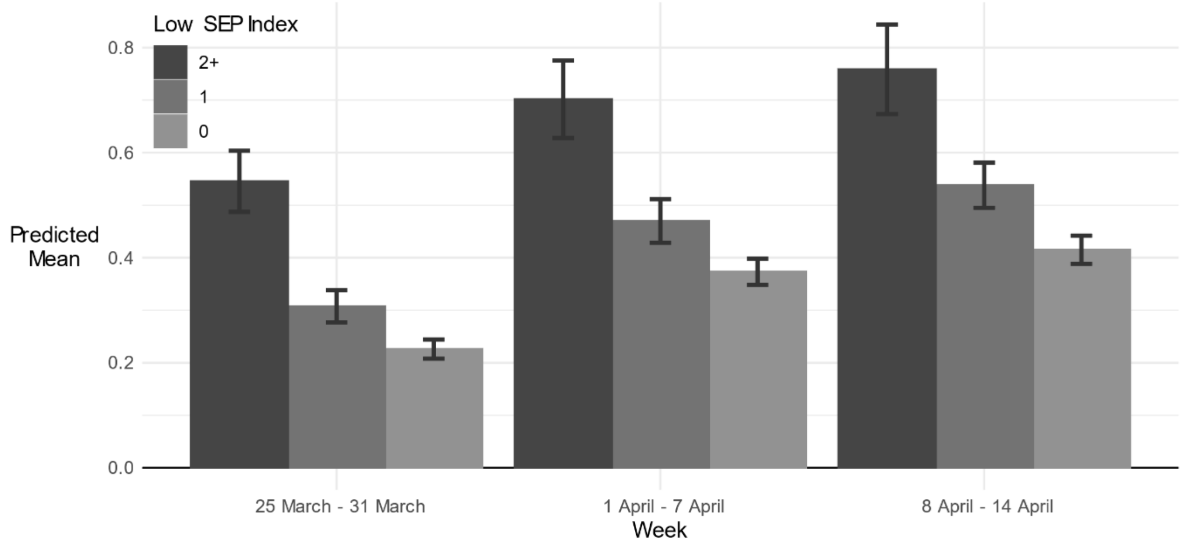

Note: Dates show the week in which adversities were reported, with reporting being on experiences in the past 7 days.

Supplementary Figure 5: Predicted probability of experiencing specific adversities by week and SEP, from fully adjusted logit models restricting the sample to those employed at baseline.

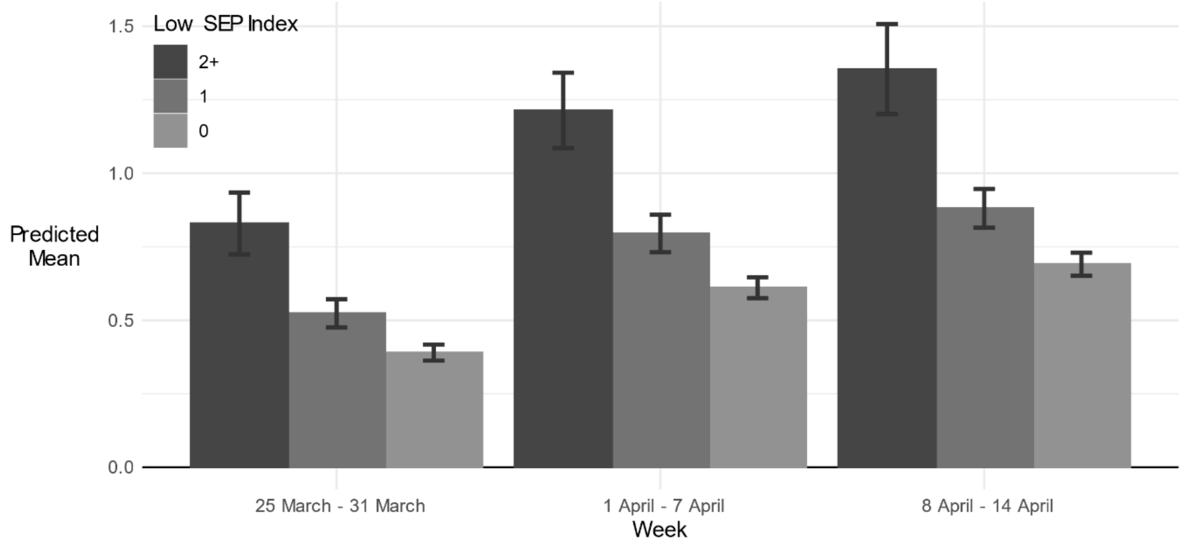

Note: Dates show the week in which adversities were reported, with reporting being on experiences in the past 7 days.
